# Special Issue: Research and Application of Food By-Products

**DOI:** 10.3390/molecules28114557

**Published:** 2023-06-05

**Authors:** Renata Różyło

**Affiliations:** Department of Food Engineering and Machines, University of Life Sciences in Lublin, Głęboka 28, 20-612 Lublin, Poland; renata.rozylo@up.lublin.pl

Recently, there has been an increase in interest in reusing food processing by-products. Such waste management is consistent with the goals of sustainable development, has an influence on the environment, and has a beneficial economic impact. Many studies have shown that waste products are a rich nutritional source and can be reused in food production or other industries. Such raw materials are frequently high in fiber, protein, minerals, and other nutrients. Furthermore, they can have a favorable impact on our health. It is vital to undertake study in this area, which is why measurements of chemical composition, phytochemical content, polyphenolic component content, bioactive compounds, and antinutritional substances are carried out. Characterization at the molecular level is also important.

Ayad et al. [1] have demonstrated that ultrasound-assisted extraction of phyto-antioxidant polyphenols from carob by-products is feasible and effective. The maximum amounts of polyphenols (TPC = 21.92 mg GAE/g d.w.), antioxidant activity (TAC = 49.30 mg AAE/g d.w.; DPPH = 90.50%), and photoprotective characteristics (SPF = 22.37) were obtained using optimal ultrasound-assisted extraction conditions (38.90% ethanol, 53.90 °C, and 50.92 min). According to research, carob can be utilized to produce ecologically friendly “green chemistry” by serving as a source of sustainable and bioactive chemicals that can be used in cosmetics.

Other research presented by Gałkowska et al. [2] has shown that blackcurrant pomace is a chemically valuable by-product whose potential can be exploited by turning pomace into food ingredients. In these tests, powdered, nonextruded, and extruded pomace was added to pasta in amounts of 5 and 10% to partially replace semolina. The presence of by-products in the pasta increased the content of total dietary fiber (from 1.89 to 10.03 g/100 g, d.w.), fat (from 1.29 to 2.70 g/100 g, d.w.), DPPH, and antiradical activity (from 253 to 1037 mol TE/g, d.w.), as well as significantly changing the color (p 0.05). The presence of the pomace also changed the texture of the cooked pasta, enhancing its firmness and hardness as well as its tensile strength when utilizing the nonextruded variant. According to the findings of these studies, durum wheat pasta enriched with 5 or 10% powdered blackcurrant pomace or their extrudates is a food product with better nutritional value and taste.

On the other hand, Sujka et al. [3] suggested buckwheat hull as an addition to pasta. The authors of this study intended to see how the partial substitution of semolina with 0, 1, 5, 10, 15, and 20% ground buckwheat hull affected the chemical composition, antioxidant properties, color, cooking characteristics, and sensory aspects of wheat pasta. Sample analyses revealed that the addition of buckwheat hull increased the fiber content from 4.31% (control pasta) to 14.15% (20% of buckwheat hull). Furthermore, as compared to the control sample, the brightness and yellowness of enriched products were dramatically reduced, with the overall color difference ranging from 23.84 (pasta with 1% BH) to 32.56 (15% buckwheat hull). Furthermore, there was a decrease in the ideal cooking time, as well as an increase in the weight index and cooking loss. The buckwheat-hull-enriched cooked pasta had significantly increased total phenolic content and antioxidant activity, although the proposed amount of this additive cannot exceed 10% due to the unpleasant flavor and odor.

In subsequent studies [4], the reuse of spinach stems as a valuable food ingredient was proposed. Wet and dry ball mill micronization, together with freeze drying, was utilized in this work to treat spinach stems and leaves to create functional powders. The micronized spinach leaf and stem powders were tested for color and particle size. GC-MS analysis was used to assess the antioxidant activity (AA) of the powders and phenolic compounds present in them. Dry micronization of the leaves and stems (d50 = 19.5 and 10.1 m, respectively) produced significantly smaller particle sizes than wet micronization (d50 = 84.6 and 112.5 m, respectively). More phenolic chemicals were recovered from the dry-micronized powders, including o-coumaric acid and gallic acid. The overall phenolic content of the stems was greatly raised by dry micronization, as was the AA of these powders. These findings show that spinach leaves and stems that have been dry-micronized can be valuable functional components of food.

Szymańska-Chargot et al. [5] proposed the use of hop stems, which are a by-product of the production of hop cones, for the production of cellulose. The thermochemical procedure was used to isolate the cellulose. After high-speed blending, the cellulose had a crystallinity degree of 67%, as determined using X-ray diffraction, and a median diameter of 6.7 nm, as determined by atomic force microscope imaging. To achieve the additional breakdown of cellulose fibers, high-intensity ultrasonication was used. The prolonged high-intensity ultrasonication treatment resulted in a reduction in crystallinity of up to 60% and a reduction in fiber diameter of up to 4 nm. The Fourier transform infrared spectroscopy (FTIR) spectra revealed that the treatment altered intermolecular hydrogen bonding.

In addition to the stems and leaves, other parts of the plant can be used, such as flowers, for example the cornflower, considered to be weeds [6]. As a result, microencapsulated red powders made from blue cornflower petals aqueous extract were proposed in additional experiments. Microencapsulation was accomplished through freeze-drying with several stabilizers, including maltodextrin, guar gum, and lecithin. The primary difference in the FT-Raman spectra was linked to a band shift, which is a reflection of the development of flavium cation forms of anthocyanins. The total phenolic content of the microencapsulated RP was 21.6–23.4 mg GAE/g d.w. and the total flavonoid content was 5.0–5.23 mg QE/g. The investigated powders’ ABTS radical scavenging activity ranged from 13.8 to 20.2 EC50 mg/mL, d.w. The powders’ reducing antioxidant power (RED) was determined to be between 31.0 and 38.7 EC50 mg/mL (d.w.), and their OH• scavenging activity ranged from 1.9 to 2.6.

A group of other researchers [7] tested the properties of biochar obtained from insect powder and chitin. The characterization and phytotoxic assessment of biochar derived from crickets and cricket chitin are presented in this paper. Cricket powder and cricket chitin were pyrolyzed at 500 and 700 degrees Celsius, respectively. The obtained biochars had a tightly “packed” solid surface/monolithic type with a nonporous structure (0.05–0.22 m^2^/g) and a high N content (9.4–11.8%), regardless of pyrolysis temperature. The biochar from cricket chitin had a higher pH (12.2–12.4) than that from cricket powder (8.7–10.8). According to the XRD results, biochars have an amorphous carbon turbostratic structure and a randomly arranged graphitic-like microcrystallite structure. The presence of multiple O_2_ and N-functional groups on the biochar surface was confirmed through FTIR spectra. Biochar made from cricket chitin was put to soil at rates ranging from 0.5 to 1.5% and greatly reduced *Lepidium sativum* germination. The thermal degradation of cricket powder and chitin stimulates the creation of organic N-containing heterocyclic rings, which results in the formation of N-doped carbons with potential use in energy storage and contaminant sorption.

The discoveries of Liu et al. [8] show a new way of using defatted Antarctic krill. This study focused on the extraction and primary characterization of antifreeze peptides from this crustacean; Protamex was shown to be the best protease for peptide production. Short peptides with MWs ranging from 600 to 2000 Da (69.2%) were discovered in this study. An amino acid composition analysis revealed that glutamic acid (18.71%), aspartic acid (12.19%), leucine (7.87%), and lysine (7.61%) were abundant. These findings show that peptides from Antarctic krill can act as a possible cryoprotectant for maintaining *L. rhamnosus*.

Chiacchio et al. [9] observed that baobab shell and fibrous filaments contain polyphenols and antioxidant dietary fibers, implying that they can be used as functional food additives. Shell, fibrous filaments, and seeds are by-products from the production of baobab pulp. The properties of these products were compared, and as a result of the tests, it was noticed that shell outperformed fibrous filaments (79%), and had the largest soluble and direct TAC (72 0.7 and 525 1.0 mol eq. Trolox/g, respectively). The polyphenol content was highest in pulp, followed by shell, fibrous filaments, and seeds. Quercetin was the most abundant polyphenol in the shell (438.7 2.5 g/g), while epicatechin was the most abundant in the pulp (514 5.7 g/g), followed by fibrous filaments (197.2 0.1 g/g), seeds (120.1 0.6 g/g), and procyanidin B_2_, which represented 26–40% of the total polyphenols in all products.

Waraczewski et al. [10] reviewed the literature on the reuse of food production waste to produce hydrocolloids. Hydrocolloids, as mentioned in this research, are naturally occurring polysaccharides or proteins that are used to gelatinize, change texture, and thicken food products, as well as in the manufacturing of edible films and medication capsules. Furthermore, various hydrocolloids, especially prebiotics high in bioactive chemicals, have been shown to benefit human health. This paper describes plant-derived hydrocolloids generated from arrowroot (*Maranta arundinacea*), kuzu (*Pueraria montana* var *lobata*), Sassafras tree (*Sassafras albidum*) leaves, sugarcane, acorn, and animal-derived gelatin. This study also mentions hydrocolloid processing and use, physicochemical activity, composition, and health advantages.

The next article, presented by Eckhardt et al. [11], mentions the possibilities of using coffee cherry (c*ascara*) by-products from coffee production, presenting an overview of the exposure and risk evaluation of several products made from coffee cherry pulp and husk, including juice, jam, jelly, puree, and flour. The authors recognized that caffeine, in particular, is regarded as a limiting factor as a bioactive element; its safe intake will be determined for various age groups, demonstrating that even adolescents could drink small amounts without detrimental health effects. Furthermore, harvesting methods and processing stages can have an impact on the composition. Most notably, dried and powdered coffee cherries can replace up to 15% of the flour in pastry items without compromising baking capabilities or sensory attributes.

A recent research work [12] shows a holistic view of the apple’s historical evolution, describes apple by-products, and examines the application of green technology to improve its functionality. The authors underlined that apples are the world’s third most extensively farmed fruit. About 30% of overall apple production is processed, with juice and cider being the main end products. This technique generates a considerable amount of apple by-products, which are often devalued and go unutilized, are landfilled, or are burnt. However, apple by-products have been shown to be a good source of bioactive elements such as dietary fiber, fatty acids, triterpenes, and polyphenols. There is potential to turn this perishable material into an attractive source of health-promoting components using HHP or assisted extraction using enzymes, supercritical fluids, microwaves, or ultrasound.

Finally, despite scientists’ keen interest in the characteristics and methods of reusing food by-products, there is an ongoing need for study in this area. Such research is required by the industry, particularly to provide innovative solutions and strategies for sustainable development that are environmentally friendly.
